# Evaluation of the understanding the degree of diabetes decompensation in elderly patients without satisfactory glycemic control

**DOI:** 10.1186/1758-5996-7-S1-A165

**Published:** 2015-11-11

**Authors:** Rafael Vaz Machry, Cibelle de Abreu Evaldt, Luthiele da Silva Vasconcellos, Rafaela Ramos Nunes, Henrique Umpierre Pedroso, Thaymê Luísa de Souza Pires, Raquel Ferreira, Eduardo Bardou Yunes Filho, Paloma Dias da Cruz, Ticiana da Costa Rodrigues

**Affiliations:** 1 Universidade Federal do Rio Grande do Sul, Porto Alegre, Brazil

## Background

The prevalence of diabetes is increasing among the elderly. In addition to the care needed to maintain appropriate blood sugar levels, it is necessary to understand the disease. Often, this population may be more difficult to understand the risks and other factors related to the disease.

## Objectives

Assess the degree of understanding of diabetes without appropriate control in elderly patients. Materials and methods: We performed a Cross study of diabetic patients in the Endocrinology Department of a Brazilian tertiary hospital who were treated between June and December 2014. We include patients over 60 yrs. of age, of both sexes, with HbA1c ≥ 8.5% using oral hypoglycemic agents and insulin. All patients underwent five questions related to satisfaction with the diet and treatment for diabetes. We reviewed the records of patients to assess the previous glycemic control.

## Results

Forty-five patients were included. Glycated hemoglobin was 10.08±0.31 (12 months before), 10.46±0.32 (six months before) and 10.34±0.22 during the interview. When asked about the lack of clear and concrete goals in diabetes care, 40% of patients responded not have problems. Another 40% considered a serious problem. The other responded intermediate response. On “feel discouraged with the treatment,” only 28% considered a serious problem and 46.7% do not consider a problem. When asked about “diet deprivation”, 20% considered a serious problem, and 48.9% did not have concerns about it. We also ask if the patients were satisfied with their current treatment. 64.4% declared satisfied, and only 4.4% reported being dissatisfied. Other answering “more or less”. The last question was about self-understanding of diabetes. 71.1% say understanding about their illness. Only 6.7% reported not being satisfied.

## Conclusion

Despite the inappropriate glycemic control, most patients do not understand the severity and stage of disease.

